# Dynamics of Time Delay-Induced Multiple Synchronous Behaviors in Inhibitory Coupled Neurons

**DOI:** 10.1371/journal.pone.0138593

**Published:** 2015-09-22

**Authors:** Huaguang Gu, Zhiguo Zhao

**Affiliations:** School of Aerospace Engineering and Applied Mechanics, Tongji University, Shanghai 200092, China; Lanzhou University of Technology, CHINA

## Abstract

The inhibitory synapse can induce synchronous behaviors different from the anti-phase synchronous behaviors, which have been reported in recent studies. In the present paper, synchronous behaviors are investigated in the motif model composed of reciprocal inhibitory coupled neurons with endogenous bursting and time delay. When coupling strength is weak, synchronous behavior appears at a single interval of time delay within a bursting period. When coupling strength is strong, multiple synchronous behaviors appear at different intervals of time delay within a bursting period. The different bursting patterns of synchronous behaviors, and time delays and coupling strengths that can induce the synchronous bursting patterns can be well interpreted by the dynamics of the endogenous bursting pattern of isolated neuron, which is acquired by the fast-slow dissection method, combined with the inhibitory coupling current. For an isolated neuron, when a negative impulsive current with suitable strength is applied at different phases of the bursting, multiple different bursting patterns can be induced. For a neuron in the motif, the inhibitory coupling current, of which the application time and strength is modulated by time delay and coupling strength, can cause single or multiple synchronous firing patterns like the negative impulsive current when time delay and coupling strength is suitable. The difference compared to the previously reported multiple synchronous behaviors that appear at time delays wider than a period of the endogenous firing is discussed. The results present novel examples of synchronous behaviors in the neuronal network with inhibitory synapses and provide a reasonable explanation.

## Introduction

Synchronization is an emerging phenomenon of a population of interacting units in nature. Synchronization has attracted great interest in many different fields such as mechanics, physics, chemistry, biology, and ecology [1, 2]. Synchronization in biological systems such as the nervous system [3–5] and cardiac myocytes [6–8] has been extensively explored. Synchronous behaviors have been observed in many central nervous systems such as the visual cortex, primary motor cortex, and somatosensory cortex [3–5], which is believed to be involved with many physiological functions including perceptual grouping, sensory motor integration, working memory, and perceptual awareness [3–5, 9, 10] as well as with brain disorders such as schizophrenia, epilepsy, and autism [9–11]. The dynamics of synchronous rhythms are related to coupling, architecture of network, endogenous electronic activity of individual neurons, and other factors such as time delay and noise [12–17].

The electrical and chemical synapses including excitatory and inhibitory synapses [18] are always considered in investigations of dynamics of neuronal networks. An increase in coupling strength of coupled neurons with electrical and/or chemical excitatory synapses can enhance synchronization [12–15]. Different from excitatory synapses, inhibitory synapses can induce more complex spatio-temporal behaviors. In general, inhibitory synapse often suppresses the activity or induces anti-phase synchronization in most investigations [19–22]. However, inhibitory synapses can induce in-phase synchronization in some conditions [20–23], e.g., slow decay inhibitory synapses can induce in-phase synchronization [20–24]. Recently, two examples indicating that fast inhibitory synapses can induce in-phase synchronization in coupled bursting neurons were investigated [25, 26]. For example, when an inhibitory neuron is introduced, anti-phase synchronization of two inhibitory coupled neurons turns into complete synchronization [25]. Information transmission delays are inherent in the nervous system due to the finite propagation speeds and time lapses occurring by both dendritic and synaptic processing [18, 27, 28], especially for the slow inhibitory synapse. Theoretically, chemical synapses can be described by nonlinear function with time delay (*τ*) [20, 28–30].

When time delay is considered, complex dynamics including in-phase synchronous behaviors in inhibitory coupled neurons have attracted significant attention [27–30]. For example, time delay can induce stable in-phase synchronization in two coupled oscillators [30–32]. Recently, the synchronous firing patterns of inhibitory coupled bursting neurons with time delays obeying a Gaussian distribution exhibit a period-adding bifurcation with the increase of the average time delay. This is an example of time delay-induced multiple synchronous behaviors [33]. However, the relationship between time delays corresponding to the synchronous behaviors and the period of the bursting remained unclear. In another investigation, inhibitory coupled neurons have been shown to exhibit multiple synchronous behaviors in motif composed of three neurons or network, and each behavior appears at time delay of odd integer multiples of half of the endogenous firing period [27, 29]. Only a single synchronous behavior appears when time delay is shorter than the period of the endogenous firing [27, 29]. The simulation result of the first synchronous behavior is consistent with the observation of synchronous behavior from biological experiment on motif composed of two neurons with slow inhibitory synapses [24].

The motif composed of two or three coupled neurons has attracted much attention [34–36] because the dynamics of the motif is a crucial step in the study of the complex phenomenon in large network [35, 36]. Further, the synchronous dynamics of motif can be well understood using the dynamics of an individual single neuron acquired fast-slow variable dissection [25], which has been widely used to analyze the dynamics of bursting and discriminate the types of bursting [37, 38]. Bursting is a complex oscillatory activity that constitutes burst containing fast spikes and quiescent state, which has been found in parts of the nervous systems including the pyramidal neurons, pyloric neurons, and thalamic neurons and plays important roles in information processing [39–41]. For example, bursting can increase reliability of synaptic transmission and might provide effective mechanisms for selective communication between neurons [40, 41].

In the present study, time delay (*τ*) induced multiple synchronous behaviors in a motif composed of three inhibitory coupled neurons with endogenous bursting when isolated are investigated. Period-*K* (*K* = 6 chosen as representative) bursting is investigated and the bursting period is labeled as *T*. Different from the results of Refs. [27, 29] wherein only the first of the multiple synchronous behaviors appears at time delays within 0 < *τ* < *T* and each of the remaining behaviors appears at time delay within (*n*−1)*T* < *τ* < *nT* (*n* = 2, 3), multiple synchronous behaviors in the present paper appear at time delay within 0 < *τ* < *T*. The multiple synchronous behaviors are period-(*K* − 2), period-(*K* − 1), period-*K* and period-(*K*+1) bursting patterns, which appear at different intervals of *τ* when coupling strength is suitable. Using bursting dynamics acquired by fast-slow variable dissection method and the response of bursting pattern to a negative impulsive current at different phase of fast spike or quiescent state, the multiple synchronous behaviors are well interpreted. An isolated neuron manifests burst with *K* − 2, *K* − 1, *K*, and *K*+1 spikes when a negative impulsive current with suitable strength is applied suitable phase within (*K* − 2)^th^, (*K* − 1)^th^, *K* ^th^ spikes, and quiescent state of a burst, respectively. For a neuron in the motif, the inhibitory coupling current received by a neuron in the motif closely resembles the negative impulsive current applied to an isolated single neuron. The time delay and the coupling strength can modulate the application time and the strength of the inhibitory coupling current, respectively. If the time delay and coupling strength is suitable, different synchronous behaviors can be induced. These results present novel synchronous behaviors induced by inhibitory synapses with time delays.

## Materials and Methods

### Leech heart interneuron model of single neuron

In fact, the multiple synchronous behaviors can be simulated in the reduced model of Leech heart interneuron, Chay model and Rulkov map model. Considering that the reduced model of Leech heart interneuron has been widely used to investigate complex dynamics of neuronal firing patterns and the spatiotemporal dynamics of neuronal motifs or networks [25, 26, 29, 34], in the present paper, Leech heart interneuron model is used as representative and is described as follows,

$$C\;\dot{V}=-[{\bar{g}}_{Na}f{\left(-150,0.0305,V\right)}^{3}h_{Na}(V-E_{Na})+{\bar{g}}_{K2}m_{K2}^{2}(V-E_{K})+g_{L}(V-E_{L})+I_{pol}]$$ (1)

$${\dot{h}}_{Na}=[f(500,0.0325,V)-h_{Na}]/\tau_{Na}$$ (2)

$${\dot{m}}_{K2}=[f(-83,0.018+V_{K2}^{shift},V)-m_{K2}]/\tau_{K2}$$ (3)

Here, *V* is the membrane potential, *h*_*Na*_ and *m*_*K*2_ are the gating variables describing the activation of potassium current and inactivation of sodium current, respectively. *C* is the membrane capacitance; ${\bar{g}}_{K2}$ and ${\bar{g}}_{Na}$ are the maximum conductance of potassium current and sodium current, respectively; *E*_*K*2_ and *E*_*Na*_ are the reversal potentials of K^+^ and Na^+^, respectively; *g*_*L*_ and *E*_*L*_ are the conductance and reversal potential of leak current, respectively; *I*_*pol*_ is the polarization current; *τ*_*Na*_ and *τ*_*K*2_ are the time constants of activation of potassium current and inactivation of sodium current, respectively; *f*(*x*,*y*,*z*) = 1 / (1 + exp{*x*(*y* + *z*)}) is a Boltzmann function describing kinetics of the currents. $V_{K2}^{shift}$ is the shift of the membrane potential of half-activation of potassium current from its canonical value.

The parameter values are as follows: *C* = 0.5 nF, ${\bar{g}}_{K2}$ = 30 nS, ${\bar{g}}_{Na}$ = 200 nS, *g*_*L*_ = 8 nS, *E*_*K*_ = −0.07 V, *E*_*Na*_ = 0.0405 V, *E*_*L*_ = −0.046 V, *I*_*pol*_ = 0.001 mA, *τ*_*Na*_ = 0.0405 s, *τ*_*K*2_ = 0.9 s.

The parameter *τ*_*K*2_ (0.9 s) is much larger than *τ*_*Na*_ (0.0405 s), therefore the model composed of Eqs (1–3) is a fast-slow system. The Eqs (1–3) and the third equation is the full system and slow subsystem (SS), respectively. The fast subsystem (FS) is the first two equations when *m*_*K*2_ is chosen as the bifurcation parameter.

### Model of the motif with time delay

The motif is composed of three identical neurons described by the reduced model of the Leech heart interneuron. The connections are reciprocal inhibition coupling and bidirectional. The model of the motif composed of three Leech heart interneurons is described as follow:

$$C\;{\dot{V}}_{i}=-[{\bar{g}}_{Na}f{\left(-150,0.0305,V_{i}\right)}^{3}h_{i}(V_{i}-E_{Na})+{\bar{g}}_{K2}m_{i}^{2}(V_{i}-E_{K})+g_{L}(V_{i}-E_{L})+I_{pol}]+I_{i}^{syn}$$ (4)

$${\dot{h}}_{i}=[f(500,0.0325,V_{i})-h_{i}]/\tau_{Na}$$ (5)

$${\dot{m}}_{i}=[f(-83,0.018+V_{K2}^{shift},V_{i})-m_{i}]/\tau_{K2}$$ (6)

Here, *h*_*i*_ and *m*_*i*_ corresponds to *h*_*Na*_ and *m*_*K*2_, respectively, and $I_{i}^{syn}$ is synaptic current received by neuron *i* (*i* = 1, 2, 3) and described as follows [29]:

$$I_{i}^{syn}=-(V_{i}(t)-E_{syn})\sum_{j=1\;(j\neq i)}^{3}g_{ij}(V_{j}(t-\tau )-\theta_{syn})$$ (7)

Here, *i*, *j* = 1, 2, 3, and *j* ≠ *i* to avoid the self-coupling. *E*_*syn*_ is the reversal potential of synaptic current. *g*_*ij*_ is the coupling strength from neuron *j* to neuron *i*. *E*_*syn*_ = −0.0625 V, which is set below the minimal value of *V*_*i*_(*t*) to ensure that the synapse is inhibitory. *θ*_*syn*_ is set as −0.03 V to ensure that every spike within a burst can cross the threshold.

### The synchronization index

A similarity function *S*_*ij*_, which has been used to investigate synchronization between neuron *i* and neuron *j* in previous studies [42], is employed in this paper and described as follows,

$$S_{ij}=\frac{<{\left(V_{i}(t)-V_{j}(t)\right)}^{2}>}{{\left[<V_{i}^{2}(t)><V_{j}^{2}(t)>\right]}^{1/2}}$$ (8)

Here, < > represent the time average, *i* = 1, 2, 3, *j* = 1, 2, 3, *j i*. *S*_*ij*_ = *S*_*ji*_. If the signals *V*_*i*_(*t*) and *V*_*j*_(*t*) are independent, the difference between them is the same order as the signals themselves. If *V*_*i*_(*t*) = *V*_*j*_(*t*), as in the case of complete synchronization, *S*_*ij*_ reaches its minimum value being as 0. In the present paper, *S*_max_ = max(*S*_12_, *S*_23_, *S*_31_) and the three neurons are regarded to achieve synchronization if *S*_max_ < 10^−4^.

### Parameter configurations

In the present paper, the coupling conductances of counterclockwise and clockwise connections are set as *g*_12_ = *g*_23_ = *g*_31_ = *g*_1_, and *g*_21_ = *g*_13_ = *g*_32_ = *g*_2_. Because of the coexistence of bursting patterns, the probability of *S*_max_ < 10^−4^ for 100 different initial values for each combination of *τ*, *g*_1_, and *g*_2_, which is labeled as *R*, is also used to characterize the synchronous dynamics.

## Results

The dynamics of period-6 bursting pattern of isolated neuron are acquired using fast-slow dissection method. The responses of period-6 bursting to a negative impulsive current applied at different phase are provided. Correspondingly, various burst patterns with different spikes per burst are induced, and the relationships between the number of spikes per burst of burst pattern and application phase and strength of the negative impulsive current are built. by the dynamics of the endogenous bursting pattern of isolated neuron, which is acquired by the fast-slow dissection method, combined with the inhibitory coupling current.

Synchronous behaviors with different bursting patterns can be simulated in inhibitory coupled neurons with time delay when different values are assigned to time delay and/or coupling strength. For a neuron in the motif, the inhibitory coupling current, of which the application time and strength is modulated by time delay and coupling strength, can play roles like the negative impulsive current. When time delay and coupling strength are suitable, single or multiple synchronous firing patterns can be induced.

### Dynamics of period-6 bursting pattern of isolated neuron

**Fig 1:**
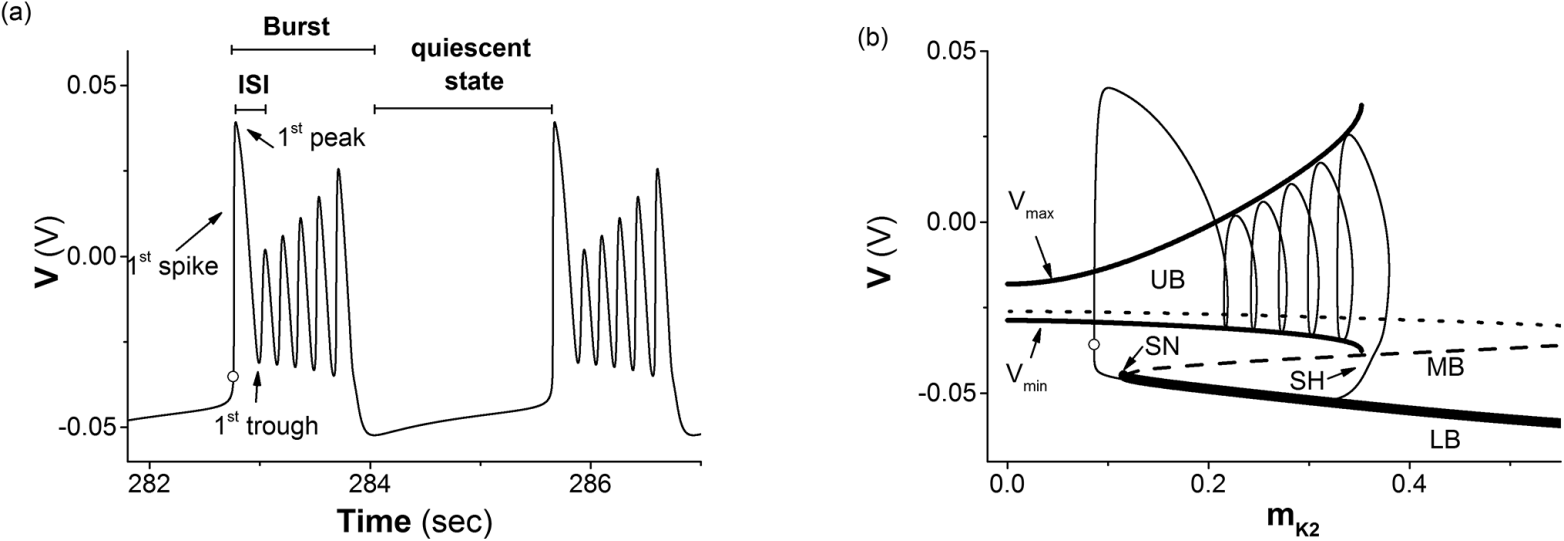
Period-6 bursting of isolated neuron when $V_{K2}^{shift}$ = −0.01 V. (a) Spike trains. The peaks of the 2^nd^ to 6^th^ spikes appear at 0.2884 s, 0.4483 s, 0.61s, 0.7761s, 0.9529 s after the cycle, and the 1^st^ to 6^th^ troughs appear at 0.233 s, 0.3959 s, 0.5612 s, 0.731 s, 0.9115 s, and 1.2788 s after the cycle; (b) the trajectory of period-6 bursting (thin solid line) in (*m*_*K*2_, *V*) plane and fast-slow variable dissection. Upper (UB, dotted line), middle (MB, dashed line), and lower (LB, bold solid line) branches of a Z-shape curve present an unstable equilibrium, saddle, and stable node of the fast subsystem. The intersection pint of MB and LB is a saddle-node (SN) bifurcation point. The upper and lower solid lines correspond to maximum (*V*_max_) and minimum (*V*_min_) values of the stable limit cycle of the fast subsystem. The intersection point of the limit cycle of the fast subsystem and the MB is a saddle-homoclinic (SH) point. Cycle: time delay is zero.

When $V_{K2}^{shift}$ = −0.01 V, the neuronal model exhibits a period-6 bursting, as shown in Fig 1(A). The period of period-6 bursting is *T* = 2.894 s.

The bifurcation structures acquired by the fast-slow dissection method are shown in Fig 1(B). The equilibrium points of the FS are composed of 3 branches, the lower branch (LB) of a stable node (bold solid line) when *m*_*K*2_ ∈ (0.1115, +∞), middle branch (MB) of a saddle (dashed line) when *m*_*K*2_ ∈ (0.1115, 0.6628), and upper branch (UB) of unstable equilibrium (dotted line) when *m*_*K*2_ ∈ (0, 0.6628). The intersection point of the LB and MB is a saddle-node (SN) bifurcation point of the equilibrium point appearing at *m*_*K*2_ = 0.1115. A stable limit cycle (LC) of which the maximal (*V*_max_ in Fig 1(B)) and minimal (*V*_min_ in Fig 1(B)) values of *V* are labeled by the upper and lower solid lines revolves around the UB when *m*_*K*2_ < 0.3523. When *m*_*K*2_ ≈ 0.3523, the LC comes into contact with the MB being as a saddle, and a homoclinic bifurcation appears. The LC becomes a homoclinic orbit and the saddle of the MB locating at the homoclinic orbit becomes a saddle-homoclinic (SH) point. The LC of the fast subsystem disappears when *m*_*K*2_ > 0.3523.

The trajectory of period-6 bursting in (*m*_*K*2_, *V*) plane of the full system with $V_{K2}^{shift}$ = −0.01 mV is illustrated by a thin solid line in Fig 1(B). With the exception of the first spike, the remaining five spikes revolve around the UB from left to right and run along *V*_max_ and *V*_min_. After the sixth spike, the trajectory runs across the MB through the neighborhood of SH and to first and then along the LB from right to left to form the quiescent state of the period-6 bursting. As the trajectory runs farther to the left and away from the SN bifurcation point, the first spike appears via a fast increase of *V*. The behaviors of period-6 bursting transit from quiescent state to spikes first via the SN (fold) bifurcation and then back to quiescent state via the homoclinic bifurcation. The bursting should be fold/homoclinic bursting [37, 38].

### The response and burst pattern of the a single neuron to a negative impulsive current

**Fig 2:**
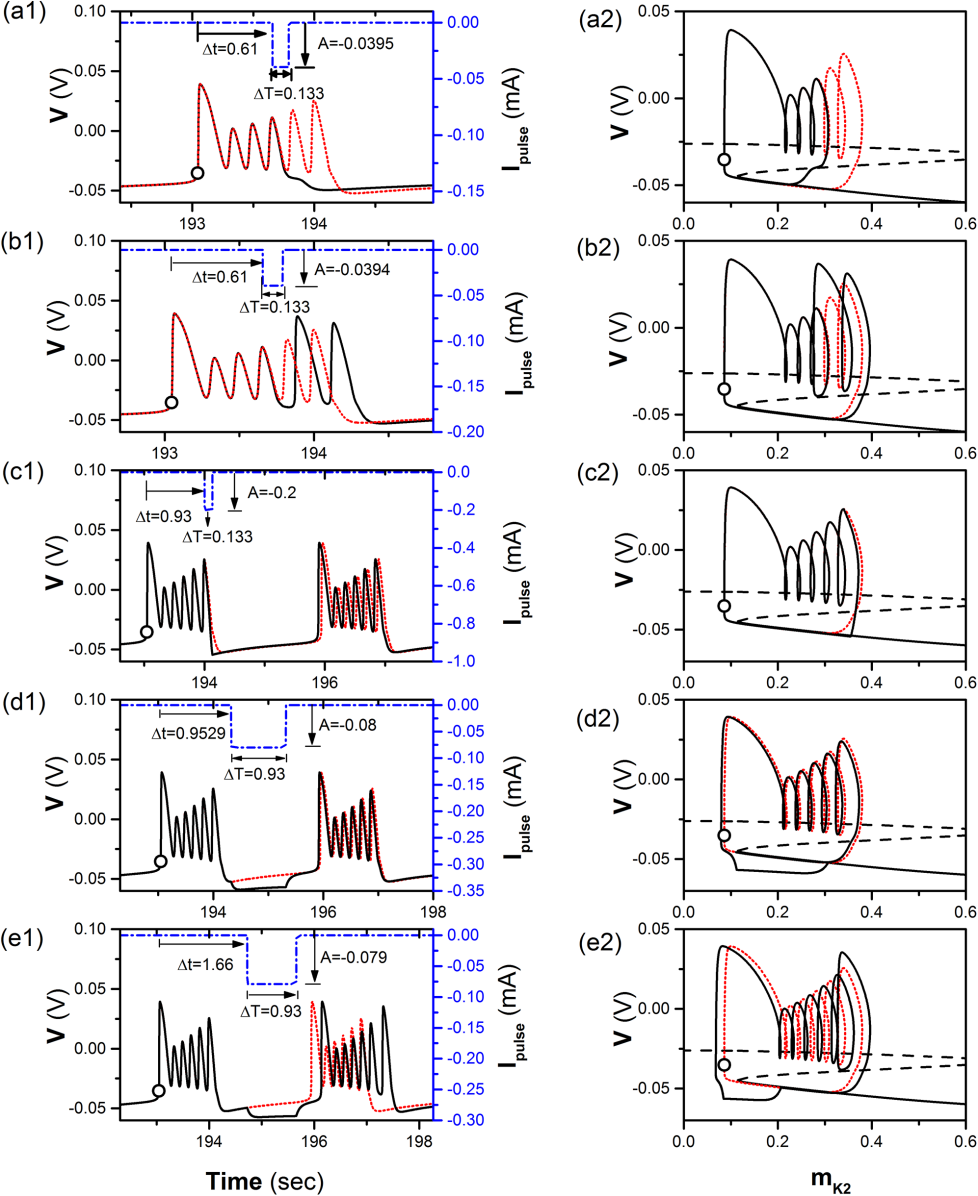
The response of a single neuron to a negative impulsive current. The burst pattern (solid line, black) induced by a negative impulsive current (dash-and-dot line, blue) with strength (*A*), width (Δ*T*) and action time (Δ*t*) and the period-6 bursting (dotted line, red) of isolated neuron. (a1) The burst with 4 spikes induced by a suprathreshold current with *A* = −0.0395 mA, Δ*T* = 0.133 s, and Δ*t* = 0.61 s; (a2) the fast-slow dissection corresponding to Fig (a1); (b1) the burst with 6 spikes induced by a subthreshold current with *A* = −0.0394 mA, Δ*T* = 0.133 s, and Δ*t* = 0.61 s; (b2) the fast-slow dissection corresponding to Fig (b1); (c1) the burst with 6 spikes induced by a current with *A* = −0.2 mA, Δ*T* = 0.133 s, and Δ*t* = 0.93 s; (c2) the fast-slow dissection corresponding to Fig (c1); (d1) the burst with 6 spikes induced by a current with *A* = −0.08 mA, Δ*T* = 0.93 s, and Δ*t* = 0.9529 s; (d2) the fast-slow dissection corresponding to Fig (d1); (e1) the burst with 7 spikes induced by a current with *A* = −0.079 mA, Δ*T* = 0.93 s, and Δ*t* = 1.66 s; (e2) the fast-slow dissection corresponding to Fig (e1). The Z-shape curve in right column present bifurcation structure of fast-slow dissection to a single neuron model.

For such a period-6 bursting, if a square “negative” pulse current with a fixed time width (labeled with Δ*T*) and strength (labeled by *A*) is introduced at a suitable time (Δ*t*), which is called action time in this paper, is measured by the time beginning from the circle shown in Fig 1(A), and lies between the *k* ^th^ peak and *k* ^th^ trough (*k* = 3, 4, 5), a burst with *k* spikes is induced when *A* is larger than a threshold, and the firing pattern is still a burst with 6 spikes when *A* is smaller than the threshold. For example, the threshold of a square “negative” current (dash-and-dot line) with Δ*T* = 0.133 s and Δ*t* = 0.61 s (the 4^th^ peak of the period-6 bursting) that can induce a burst with 4 spikes (solid line) is *A* = −0.0395 mA, as shown in Fig 2(A1). The dotted line in Fig 2(A1) represents the period-6 bursting. The trajectory of the burst with 4 spikes (solid line) and the period-6 bursting (dotted line) is show in Fig 2(A2). The trajectory can run across MB and switch to LB; therefore, the expected burst with 6 spikes (dotted line) can be changed to burst with 4 spikes. If the strength is subthreshold, for example, *A* = −0.394 mA, the burst still contains 6 spikes, as shown in Fig 2(B1). The trajectory does not cross MB, as shown in Fig 2(B2).

**Fig 3:**
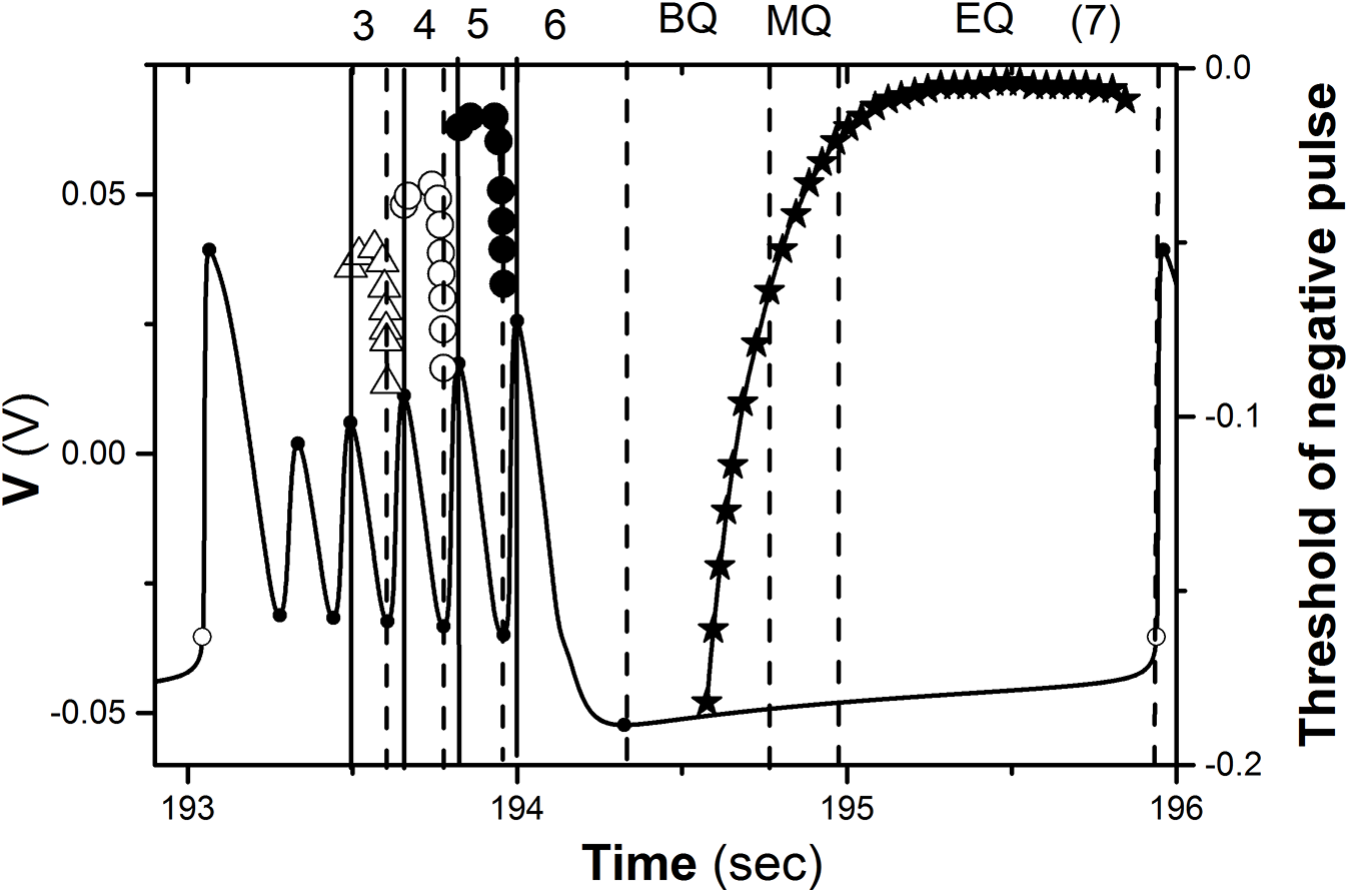
Threshold of negative impulsive current applied at different phase of period-6 bursting. The numbers 3, 4, 5, and 6 lying between vertical solid and dashed lines represent burst with 3, 4, 5, and 6 spikes, respectively. The number 7 represent burst with 7 spikes. BQ, MQ, and EQ lying between two dashed lines represent the beginning, middle, and end of the quiescent state of period-6 bursting, respectively. Period-6 bursting is shown by solid line.

If action time Δ*t* is changed from the *k* ^th^ peak to (*k* + 1)^th^ trough, the threshold of negative current with Δ*T* = 0.133 s decreases first and then increases drastically, as shown by the lines with triangle (*k* = 3), circle (*k* = 4), and dot (*k* = 5) in Fig 3. The threshold increases as *k* is decreased from 5 to 3, as shown in Fig 3. If Δ*t* is between *k* ^th^ trough and (*k* + 1)^th^ peak (*k* = 3, 4, 5), i.e., the ascending branch, it is much more difficult to induce a burst different from the burst with 6 spikes than Δ*t* between *k* ^th^ peak and *k* ^th^ trough, i.e. the descending branch.

When Δ*t* of a square “negative” current with Δ*T* = 0.133 s is between the 6^th^ peak and 6^th^ trough, the firing is still bursting with 6 spikes per burst when *A* is chosen as a value from 0 to a large enough value. The threshold of the negative current to induce a burst with 6 spikes is zero. Examples with *A* = −0.2 mA are shown in Fig 2(C1) and 2(C2). The quiescent state of period-6 bursting can be divided into 3 sub-regions, the beginning (BQ), middle (MQ), and end (EQ) of the quiescent state. When Δ*t* of a square “negative” current with Δ*T* = 0.93 s is within the beginning sub-region of the quiescent state (1.28 s Δ*t* 1.71 s), the firing is still burst with 6 spikes even if *A* is very strong and the threshold of the negative impulse to induce firing differently from period-6 bursting is shown by the line with a star. The example with *A* = −0.08 mA and Δ*t* = 0.9529 s is shown in Fig 2(D1) and 2(D2).

When Δ*t* is within the middle (1.70 s Δ*t* 1.93 s) and end (1.94 s Δ*t* 2.88 s) of the quiescent state, a square “negative” current with Δ*T* = 0.93 s can induce a burst with 7 spikes (solid line) when *A* is larger than a threshold, and the firing is still burst with 6 spikes when *A* is smaller than the threshold. The example of Δ*t* = 1.70 s is shown in Fig 2(E1) and 2(E2) and the threshold is −0.079 mA. After the negative impulsive current, the membrane potential decreases to a large extent firstly, and then the trajectory of the first spike moves to left to form a burst with 7 spikes.

With increasing Δ*t*, the change of the threshold of the negative current to induce a burst with 7 spikes decreases, as shown in Fig 3. The threshold within the middle sub-region of the quiescent state is relatively large and decreases. The threshold within the end sub-region of the quiescent state remains at a roughly fixed level, which is less than that to induce a burst with 5 spikes and is larger than that to induce a burst with 6 spikes when Δ*t* is within a descending branch of the 6^th^ spike and within the beginning sub-region of the quiescent state.

**Fig 4:**
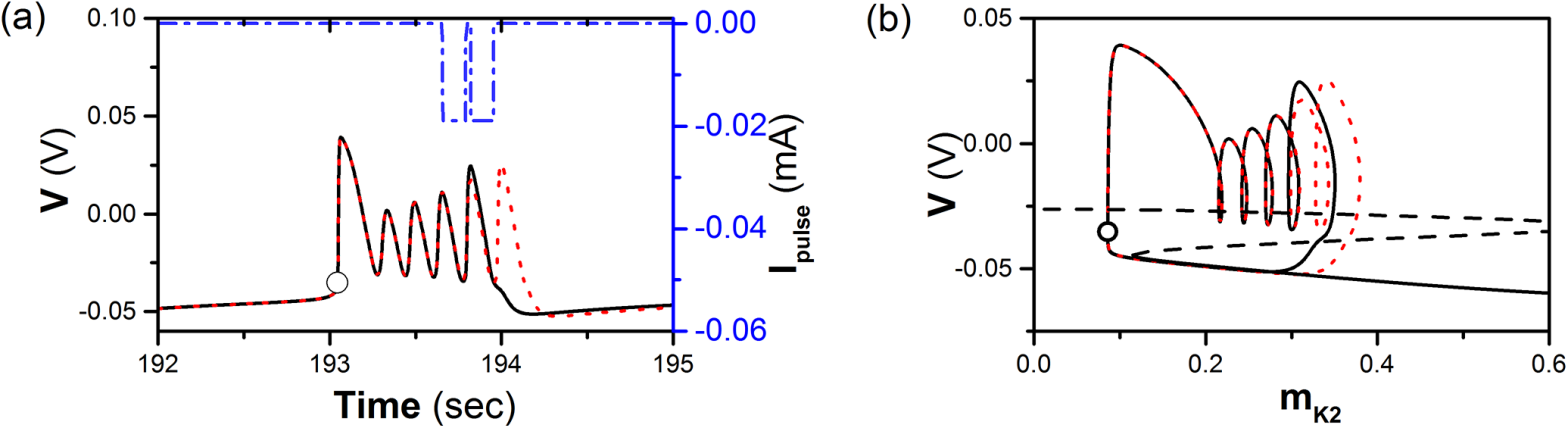
The response of a pair of negative impulses on a single neuron model. Burst with 5 spikes (solid line, black) is induced by a pair of negative impulsive currents (dash-and-dot line, blue). Period-6 bursting is shown by thin dashed line (red). (a) *A* = 0.019 mA and Δ*T* = 0.133 s. Δ*t* of the former and latter impulses is 0.61 s (within 4^th^ spike) and 0.7761 s (within 5^th^ spike), respectively; (b) Fast/slow dissection corresponding to Fig 4(a). The Z-shape curve presents bifurcation structure of fast-slow dissection to a single neuron model.

With exception of the single “negative” impulse, a pair of “negative” impulses is also considered. If Δ*t* of a subthreshold square “negative” impulse current is within *k* ^th^ spike and Δ*t* of a suprathreshold current is within (*k* + 1)^th^ spike (*k* = 3, 4, 5), a burst with *k* + 1 spikes can be evoked. For example, the behavior when *k* = 4 is shown in Fig 4. The doublet impulses, the burst with 5 spikes, and the period-6 burst are shown by dash-and-dot, dotted, and solid lines, respectively.

### Multiple synchronous behaviors and the coexisting firing patterns of the motif

**Fig 5:**
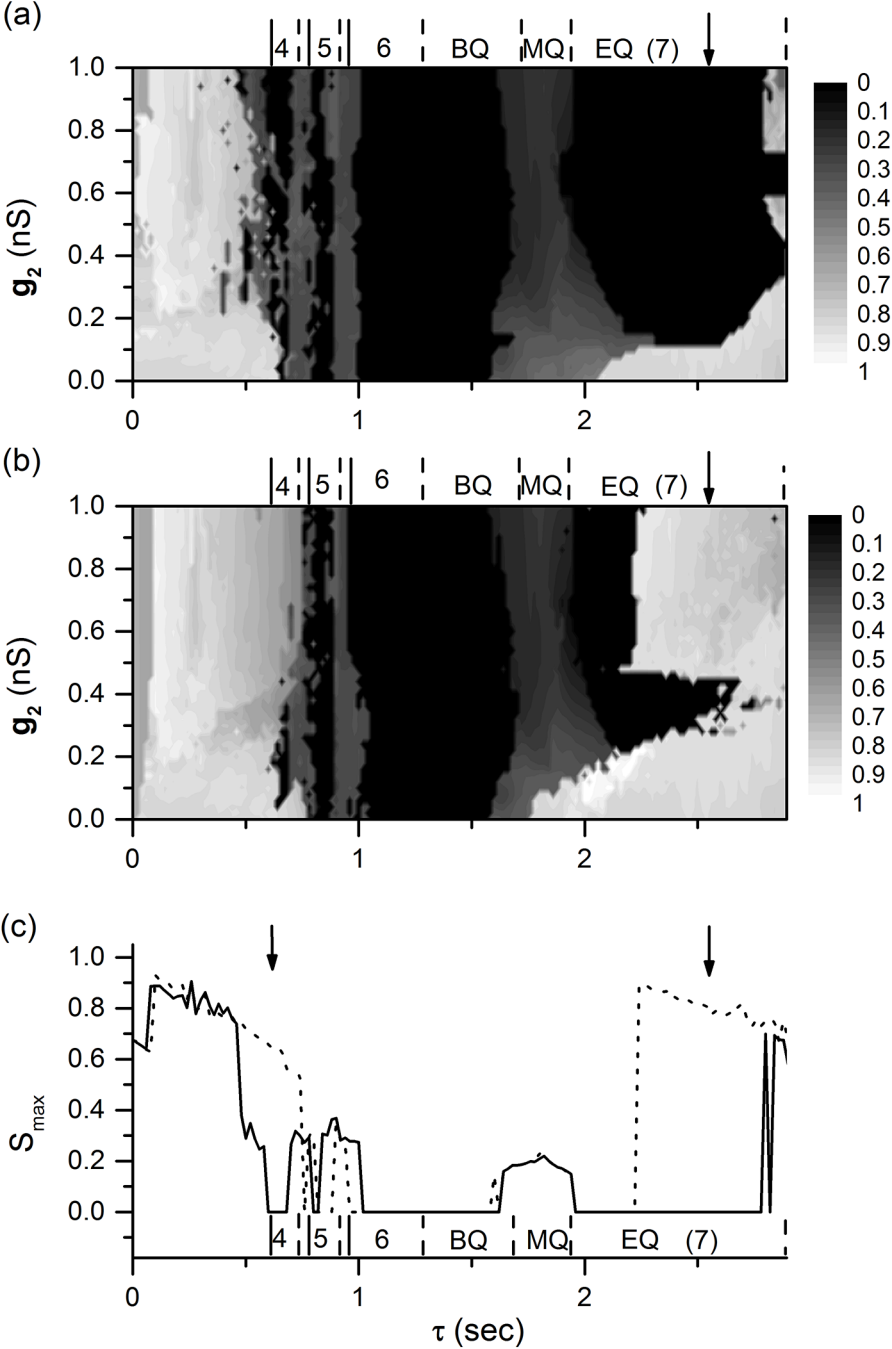
Multiple synchronous behaviors and coexistence of firing patterns. The numbers 3, 4, 5, 6, and 7 represent burst with 3, 4, 5, 6, and 7 spikes, respectively. BQ, MQ, and EQ lying between two dashed lines represent the beginning, middle, and end of the quiescent state of period-6 bursting (bursting pattern of isolated neuron), respectively. (a) The dependence of *S*_max_ on both *g*_2_ and *τ*; (b) the dependence of *S*_max_ on both *g*_2_ and *τ* with initial values different from those of Fig (a); (c) the dependence of *S*_max_ on *τ* when *g*_1_ = 0.6 nS and *g*_2_ = 0.9 nS. The dotted (solid) line corresponds to initial values of Fig 5(a) (Fig 5(b)).

When values of parameter *g*_1_, *g*_2_, and *τ* are suitable, the motif can exhibit multiple synchronous behaviors and the coexisting firing patterns. For example, when *g*_1_ = 0.6 nS, the distribution of *S*_max_ values in the plane (*τ*, *g*_2_) for two different initial values are illustrated in Fig 5(A) and 5(B), respectively. The synchronous behaviors (*S*_max_ < 10^-4^) can be found in multiple ranges of 0 < *τ* < *T*, as shown by the arrows in Fig 5(A) and 5(B). The positions of arrows from left to right correspond to those of the lines shown in Fig 3. The multiple synchronous behaviors mainly appear when 0 < *τ* < *T*, which is different from those in Refs. [23, 38], wherein each of multiple synchronous behaviors appear within the range of (*n*−1)*T* < *τ* < *nT* (*n* = 1, 2, 3,), and the only one synchronous behavior appears within the range of 0 < *τ* < *T*.

**Fig 6:**
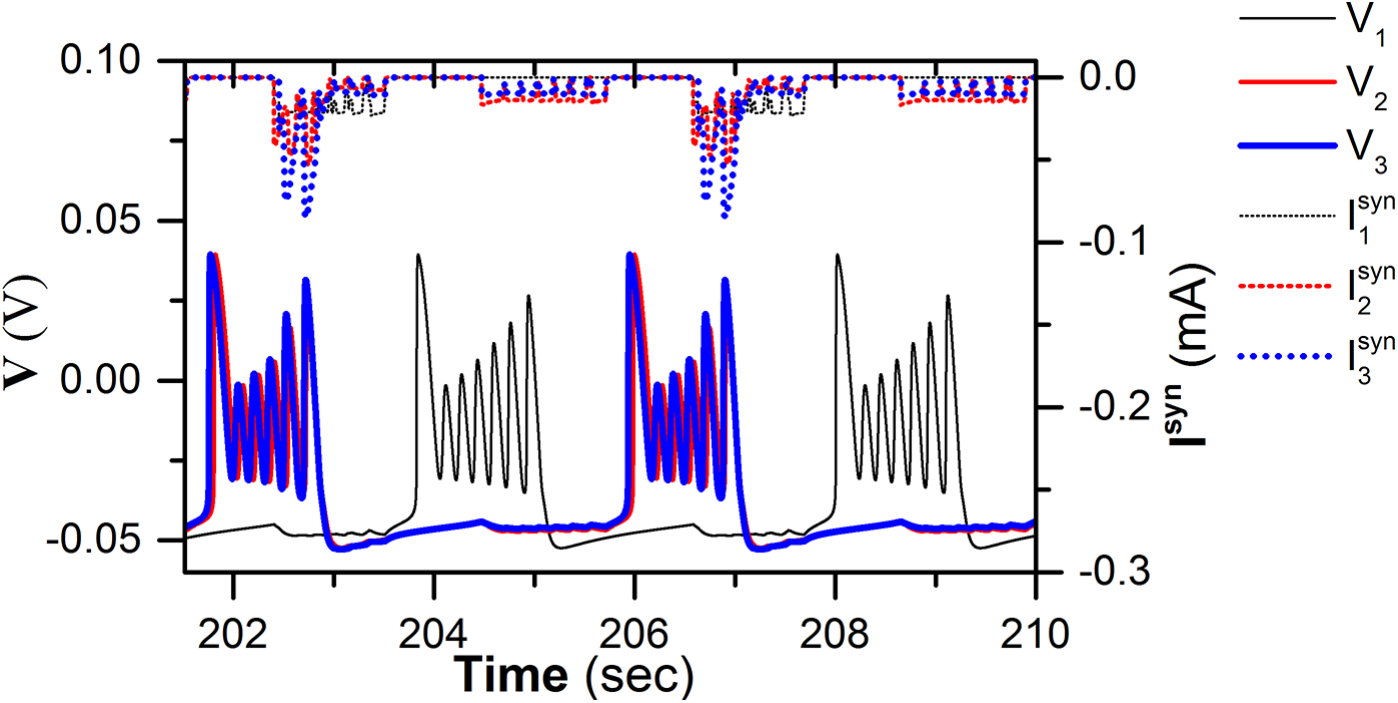
Non-synchronous bursting pattern. Membrane potential of neuron 1 (thin solid line, black), 2 (middle solid line, red), and 3 (bold solid line, blue), and coupling current of neuron 1 (thin dotted line, black), 2 (middle dotted line, red), and 3 (bold dotted line, blue) when *τ* = 0.65 s, *g*_1_ = 0.6 nS, and *g*_2_ = 0.9 nS.

**Fig 7:**
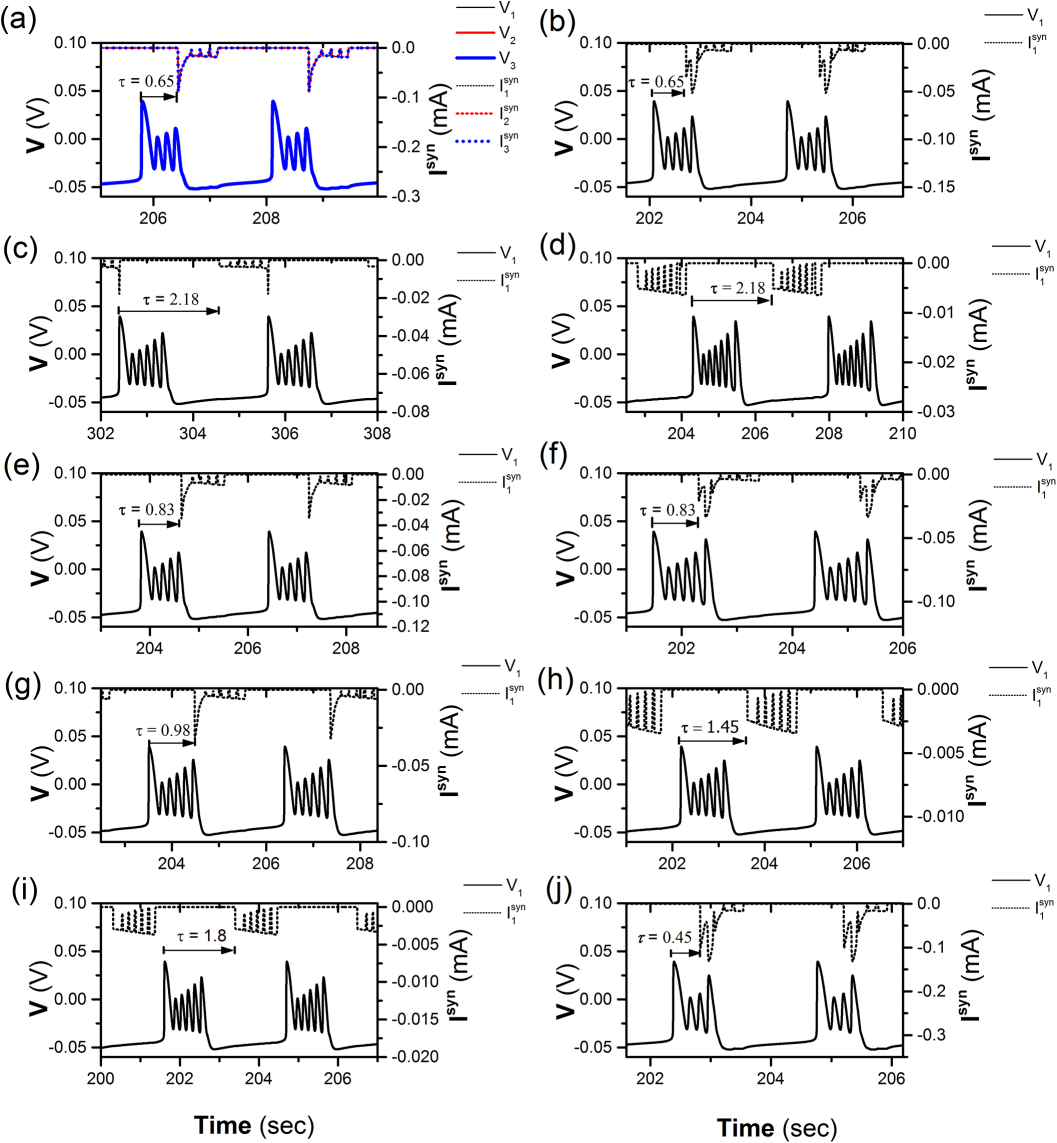
Different synchronous bursting patterns at different values of (*τ*, *g*_1_, *g*_2_). The spike trains (solid line) and the corresponding coupling current (dotted line). (a) $P_{4}^{4}$ pattern (0.65s, 0.6 nS, 0.9 nS) of neuron 1 (thin), 2 (middle), and 3 (bold); Only spike trains and coupling current of neuron 1 is shown in Figs (b)-(j): (b) $P_{4}^{5}$ pattern (0.65 s, 0.2 nS, 0.4 nS); (c) $P_{qe}^{7}$ pattern (2.18 s, 0.2 nS, 0.16 nS); (d) $P_{qe}^{6}$ pattern (2.18 s, 0.01 nS, 0.2 nS); (e) $P_{5}^{5}$ pattern (0.83 s, 0.2 nS, 0.4 nS); (f) $P_{5}^{6}$ pattern (0.83 s, 0.2 nS, 0.16 nS); (g) $P_{6}^{6}$ pattern (0.98 s, 0.001 nS, 0.42 nS); (h) $P_{qb}^{6}$ pattern (1.45 s, 0.01 nS, 0.2 nS); (i) $P_{qm}^{6}$ pattern (1.8 s, 0.01 nS, 0.2 nS); (j) $P_{3}^{4}$ pattern (0.45 s, 0.6 nS, 0.9 nS). For $P_{l}^{k}$ (*l* = 3, 4, 5 or 6, *k* = 4, 5 or 6) and $P_{qc}^{k}$ (*k* = 6 or 7, “c” represents ‘‘b‘‘, “m” or “e”, respectively), the numbers 3, 4, 5, 6, and 7 of superscript represent synchronous burst with 3, 4, 5, 6, and 7 spikes, respectively. The numbers 3, 4, 5, 6 of subscript show that action positions of coupling current are within 3th, 4th, 5th, and 6^th^ spikes, respectively. The characters “b”, “m” or “e” of subscript show that action positions of coupling current are within beginning, middle, and ending part of the quiescent state of period-6 bursting, respectively.

The difference can be found between Fig 5(A) and Fig 5(B). For example, the value of *S*_max_ shown by the bold arrows in the upper and lower panels is approximately zero and 1, respectively, showing the coexistence of different behaviors at some *τ* values. When *g*_1_ = 0.6 nS and *g*_2_ = 0.9 nS, the changes of *S*_max_ values with respect to *τ* for the initial values corresponding to Fig 5(A) and 5(B) are shown by the solid and dotted lines in Fig 5(C). Distinction between the two lines, i.e., the coexisting behaviors, can be found, as shown by two arrows in Fig 5(C). When *τ* = 0.65 s (left arrow), the dotted line presents a non-synchronous bursting pattern with *S*_max_ = 0.645 (Fig 6), and the solid line presents the coexisting synchronous period-4 bursting with *S*_max_ = 0 (Fig 7(A)).

The spike trains of the period-4 bursting patterns of three neurons illustrated in Fig 7(A) are shown by solid lines, which show synchronous behaviors. The coupling currents of the three neurons are shown by dotted lines in Fig 7(A). There was a negative impulse of the coupling current that is located near the 4^th^ spike and is suprathreshold to induce a burst with 4 spikes per burst, which is similar to that shown in Fig 2(A).

Except for the synchronous period-4 bursting, many synchronous bursting patterns can be simulated with different combinations of *τ*, *g*_1_, and *g*_2_ values when 0 < *τ* < *T*, 0 < *g*_1_ < 1, and 0 < *g*_2_ < 1, as shown in Fig 7(B)–7(J). For convenience, only spike trains and coupling currents of neuron 1 are shown in Fig 7(B)–7(J). For example, if the coupling strength is decreased from the values of Fig 7(A) to *g*_1_ = 0.2 nS and *g*_2_ = 0.4 nS, the negative impulse of the coupling current (dotted line) at the 4^th^ spike becomes subthreshold while the negative impulse at the 5^th^ spike becomes suprathreshold, a burst with 5 spikes is induced, as shown in Fig 7(B). This condition is similar to that of the double impulses shown in Fig 4. The former impulse is subthreshold and the latter is suprathreshold. When *τ* is lengthened to 2.18 s, *g*_1_ = 0.2 nS, and *g*_2_ = 0.16 nS, bursts with 7 spikes are induced by the suprathreshold “negative” current of the coupling current, as shown in Fig 7(C), which is similar to Fig 2(E). If the coupling strength is decreased to *g*_1_ = 0.01 nS and *g*_2_ = 0.2 nS, the coupling current becomes subthreshold and the synchronous bursting pattern with 6 spikes appears, as shown in Fig 7(D).

### Definition of different synchronous bursting patterns

Based on the relationships between the synchronous firing pattern and the corresponding coupling current, $P_{l}^{k}$ or $P_{qc}^{k}$ is used to discriminate different synchronous patterns. For example, the synchronous bursting shown in Fig 7(A), 7(B), 7(C), and 7(D) are $P_{4}^{4}$, $P_{4}^{5}$, $P_{qe}^{7}$, and $P_{qe}^{6}$, respectively. For both $P_{l}^{k}$ and $P_{qc}^{k}$, “*k*” presents the number of spikes per burst of the synchronous firing pattern (*k* = 4, 5, 6, 7). For $P_{l}^{k}$, “*l*” is an integer number, which shows that the delayed coupling “inhibitory” current is at *l* ^th^ spike of the burst (*l* = 3, 4, 5, 6). For $P_{qc}^{k}$, “*q*” means that the action time of delayed coupling current is within the quiescent state, and “*c*” is a character of “b”, or “m”, or “e”, corresponding to the beginning, middle and end sub-regions of the quiescent states. Therefore, the synchronous bursting patterns shown in Fig 6E–6J are $P_{5}^{5}$, $P_{5}^{6}$, $P_{6}^{6}$, $P_{qb}^{6}$, $P_{qm}^{6}$, and $P_{3}^{4}$, respectively, which closely match the examples analyzed in Fig 2.

### The dependence of the synchronous behaviors on coupling strength and time delay

**Fig 8:**
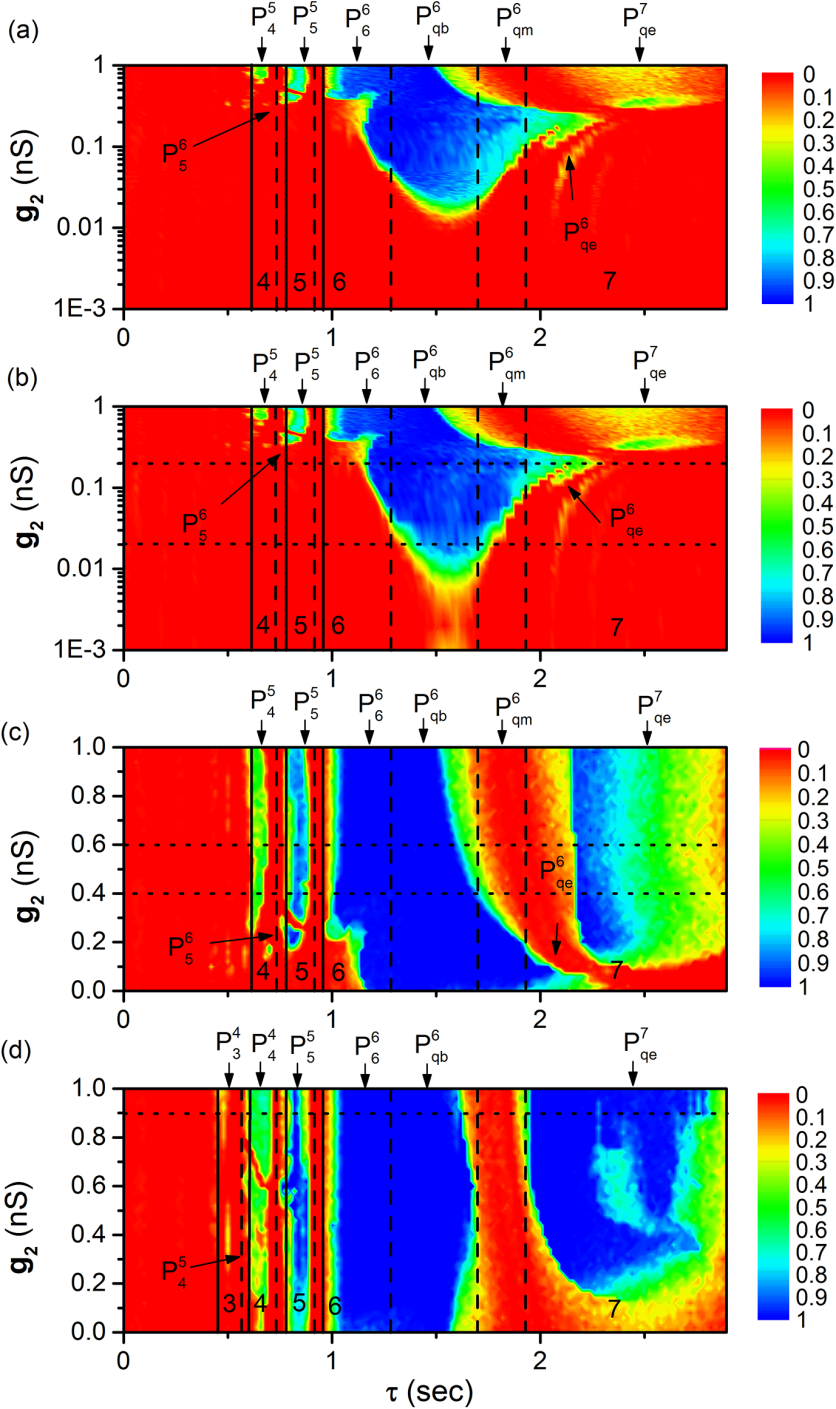
Time-delay (*τ*)-induced multiple synchronous behaviors. The distribution of *R* on plane (*τ*, *g*_2_) at different *g*_1_ levels. The numbers 3, 4, 5, 6, and 7 represent bursting pattern with 3, 4, 5, 6, and 7 spikes, respectively. $P_{l}^{k}$ (*l* = 3, 4, 5 or 6, *k* = 4, 5 or 6) and $P_{qc}^{k}$ (*k* = 6 or 7, “c” represents “b”, “m” or “e”, respectively) represent synchronous bursting patterns shown in Fig 7. (A) *g*_1_ = 0.001 nS; (b) *g*_1_ = 0.01 nS; (c) *g*_1_ = 0.2 nS; (d) *g*_1_ = 0.6 nS.

Multiple synchronous behaviors in detail and the relationships between different synchronous bursting patterns can be further found in the distributions of *R* in the two-dimensional parameter space (*τ*, *g*_2_) at four different levels of *g*_1_ = 0.001 nS, 0.01 nS, 0.2 nS, and 0.6 nS, as shown in Fig 8(A)–8(D), respectively.

Multiple synchronous behaviors appear at multiple *τ* ranges when 0 < *τ* < *T* and coupling strength is relatively strong, as shown in Fig 8(C) and 8(D), and in the upper part of Fig 8(A) (*g*_2_ > 0.3 nS) and (b) (*g*_2_ > 0.29 nS). The multiple synchronous behaviors are different from the previous studies, wherein the multiple synchronous bursting patterns appear, and each synchronous pattern is located within (*n*−1)*T* < *τ* < *nT* (*n* = 1, 2, 3). When the coupling strength is weak, for example, 0.011 nS *g*_2_ 0.3 nS in the Fig 8(A) and 0.001 nS *g*_2_ 0.29 nS in the Fig 8(B), the synchronous behavior appears only once within 0 < *τ* < *T*, and the synchronous bursting pattern is $P_{qb}^{6}$. The results also show that single synchronous behavior is changed to multiple synchronous behaviors within 0 < *τ* < *T* with increasing coupling strength, as shown in Fig 8.

The relationships between various synchronous patterns can be clearly found. For example, the coupling strength of $P_{4}^{4}$, $P_{5}^{5}$, and $P_{qe}^{7}$ is larger than that of $P_{4}^{5}$, $P_{5}^{6}$, and $P_{qe}^{6}$. From left to right, the synchronous bursting patterns with 4, 5, 6, and 7 spikes per burst appear. The *τ* ranges of bursting patterns with 6 spikes and with 7 spikes per burst are much wider than bursting patterns with 4 and 5 spikes per burst, which is consistent with the width of the curves of the threshold values shown in Fig 3.

The synchronous bursting patterns with 6, 7, 5, 4 and 3 spikes per burst appear in sequence with increasing *g*_2_, which is consistent with the values of the threshold curve shown in Fig 3. With increasing coupling strengths *g*_1_ and *g*_2_, the probabilities *R* of the same synchronous patterns increase or remain nearly 1.0, as shown in Fig 8.

**Fig 9:**
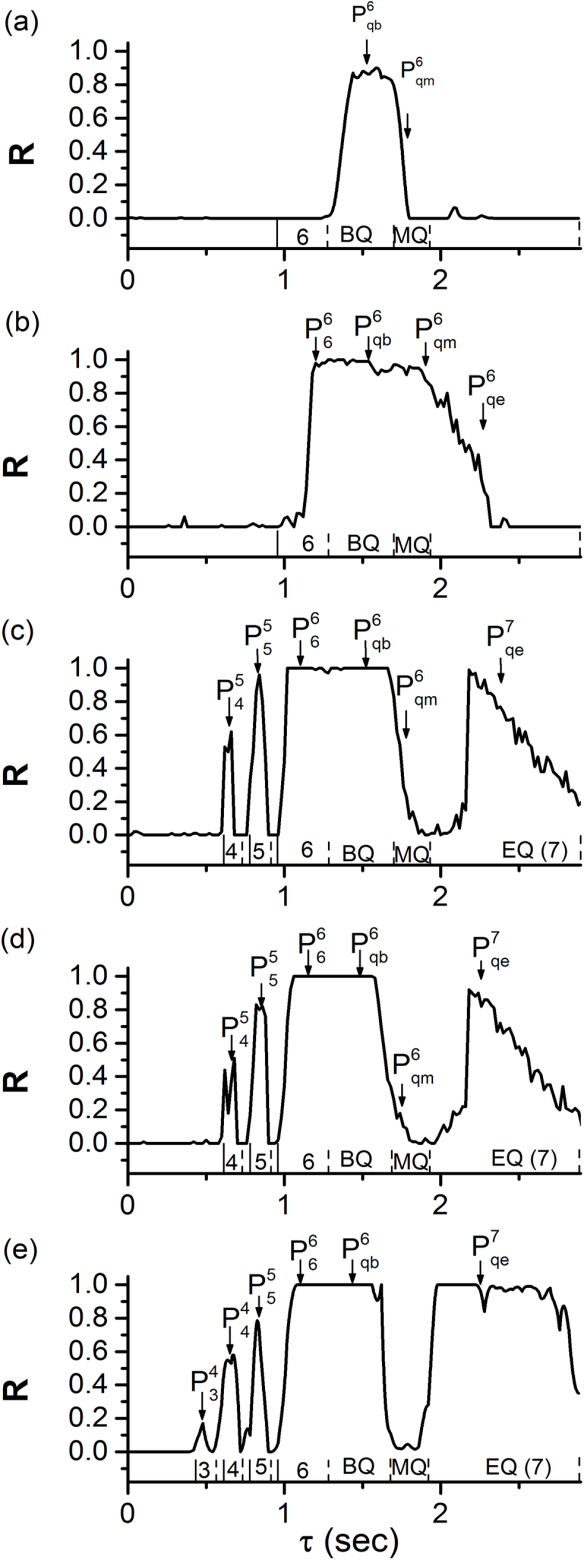
Time-delay (*τ*)-induced single or multiple synchronous behaviors. The changes of *R* values with respect to *τ* at different coupling strengths (*g*_1_,*g*_2_). The numbers 3, 4, 5, 6, and 7 represent burst with 3, 4, 5, 6, and 7 spikes, respectively. BQ, MQ, and EQ represent the beginning, middle, and end of the quiescent state of period-6 bursting, respectively. $P_{l}^{k}$ (*l* = 3, 4, 5 or 6, *k* = 4, 5 or 6) and $P_{qc}^{k}$ (*k* = 6 or 7, “c” represents “b”, “m” or “e”, respectively) represent synchronous bursting patterns shown in Fig 7. (a) Single synchronous behaviors (0.01 nS, 0.02 nS); (b) (0.01 nS, 0.2 nS); (c) (0.2 nS, 0.4 nS); (d) (0.2 nS, 0.6 nS); (e) (0.6 nS, 0.9 nS). Multiple synchronous behaviors are shown in Figs (b)-(e).

The changes of *R* with respect to *τ* along the five dotted lines in Fig 8(A)–8(D) manifest the transition from single synchronous behavior to multiple synchronous behaviors with increasing (*g*_1_,*g*_2_), as shown in Fig 9.

### The dependence of *R* on *g*_1_ and *g*_2_ at different *τ* levels

**Fig 10:**
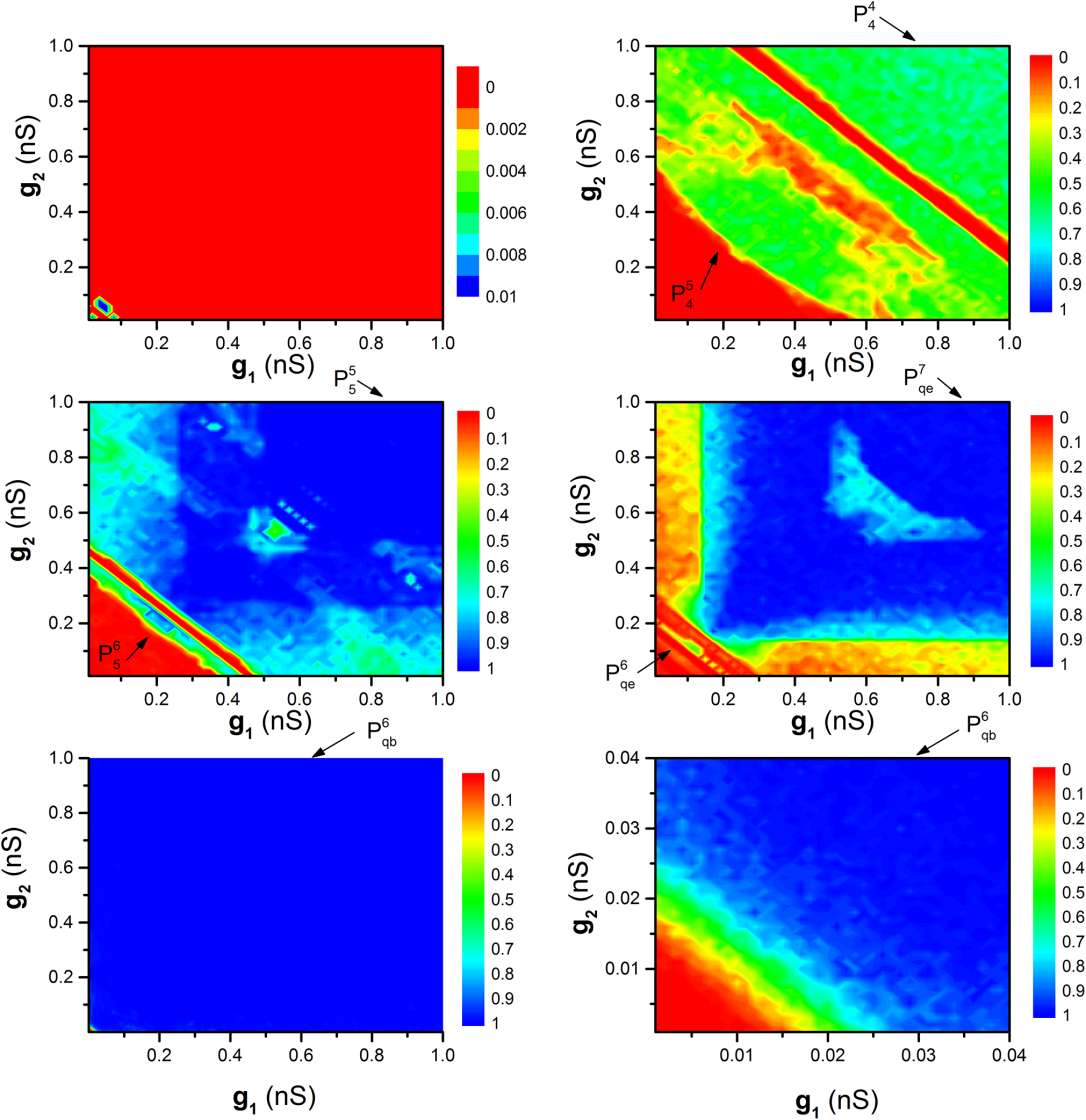
The dependence of *R* on *g*_1_ and *g*_2_ at different *τ* values. $P_{l}^{k}$ (*l* = 4, 5 or 6, *k* = 4, 5 or 6) and $P_{qc}^{k}$ (*k* = 6 or 7, “c” represents “b” or “e”) represent synchronous bursting patterns. (a) *τ* = 0 s; (b) *τ* = 0.65 s; (c) *τ* = 0.83 s; (d) *τ* = 2.3 s; (e) *τ* = 1.45 s; (f) Enlargement of (e).

The distribution of *R* values in the two-dimensional parameter space (*g*_1_, *g*_2_) at different *τ* levels is helpful to understand the influence of the cooperation between *g*_1_ and *g*_2_ on the synchronization dynamics, as shown in Fig 10. Five levels of *τ*, *τ* = 0 s (Fig 10(A)), *τ* = 0.65 s (Fig 10(B)), *τ* = 0.83 s (Fig 10(C)), *τ* = 2.3 s (Fig 10(D)), *τ* = 1.45 s (Fig 10(E) and 10(F)), are investigated as representatives.

When *τ* = 0, most *R* values equal 0 and the remaining equal 0.01, indicating that the motif is difficult to achieve synchronization, as shown in Fig 10(A). When *τ* ≠ 0, the *R* values exhibit symmetrical characteristics with *g*_1_ = *g*_2_ in the (*g*_1_, *g*_2_) plane, showing that *g*_1_ and *g*_2_ play equivalent roles in influencing the synchronization dynamics, as shown in Fig 10B–10F. When *τ* = 0.65 s, *τ* = 0.83 s, and *τ* = 2.3 s, two separated regions are shown by arrows in Fig 10(B), 10(C), and 10(D), respectively. The lower left region is $P_{4}^{5}$, $P_{5}^{6}$, and $P_{qe}^{6}$, and right upper region is $P_{4}^{4}$, $P_{5}^{5}$, and $P_{qe}^{7}$, which is consistent with Fig 8. It should be noted that the motif exhibits synchronization when the sum of *g*_1_ and *g*_2_ are strong. When *τ* 1.45 s, there is only one synchronous bursting pattern ($P_{qb}^{6}$), as shown in Fig 10(E) and 10(F) (Fig 10(F) is the enlargement of the down-left corner).

When comparing Fig 10(B)–10(D) with Fig 10(F), it can be found that the threshold of coupling strengths that can induce synchronous behaviors of $P_{q}^{6}$, $P_{qe}^{7}$, $P_{5}^{5}$ and $P_{4}^{4}$ increases in sequence, which is consistent with that of Fig 8.

### The multiple synchronous behaviors of the motif composed of neurons with other bursting patterns

**Fig 11:**
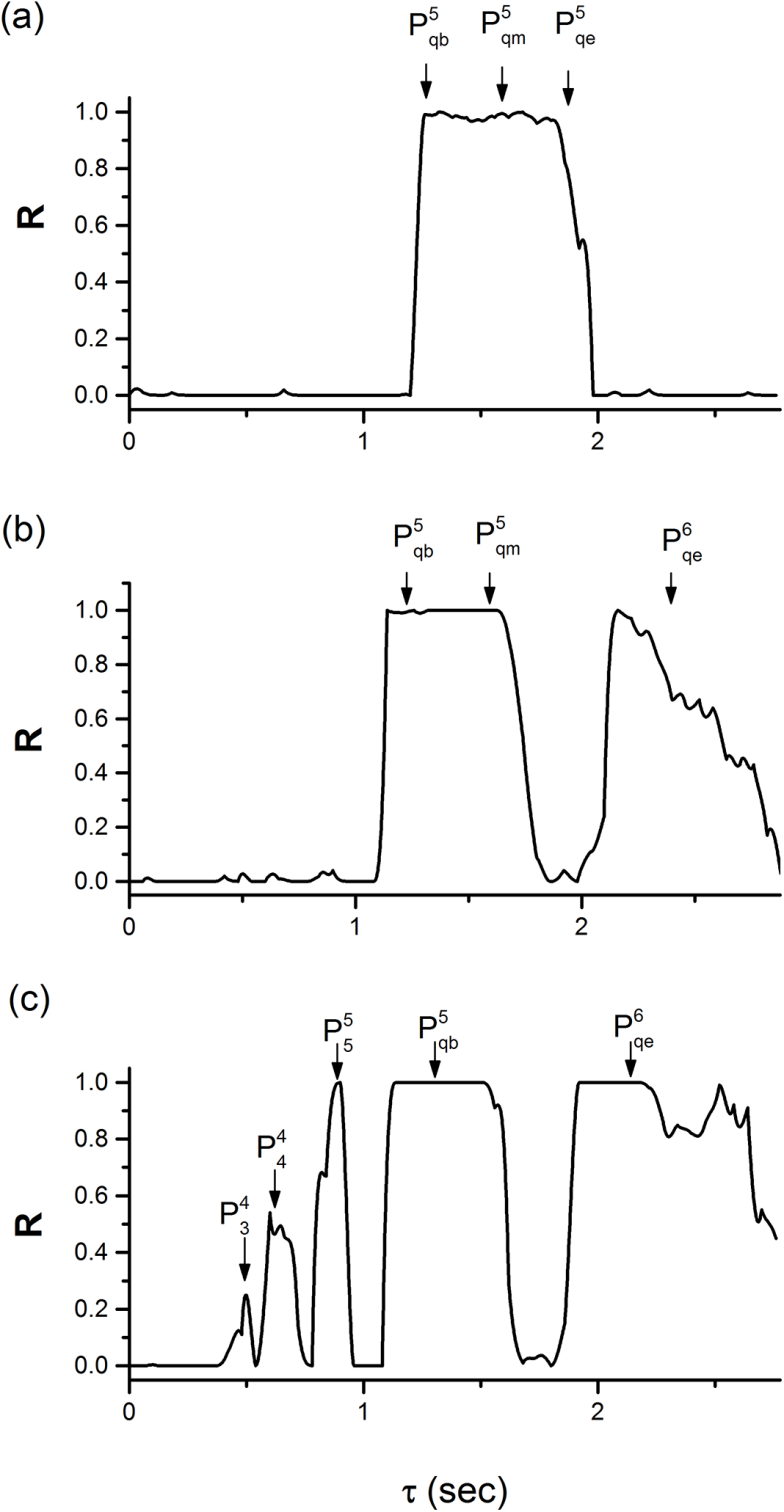
Single or multiple synchronous behaviors at different coupling strengths. The behavior of the neuron in the motif is period-5 bursting with $V_{K2}^{shift}$ = −0.008 V. (a) *g*_1_ = 0.2 nS and *g*_2_ = 0.16 nS; (b) *g*_1_ = 0.3 nS and *g*_2_ = 0.18 nS; (c) *g*_1_ = 0.6 nS and *g*_2_ = 0.6 nS.

**Fig 12:**
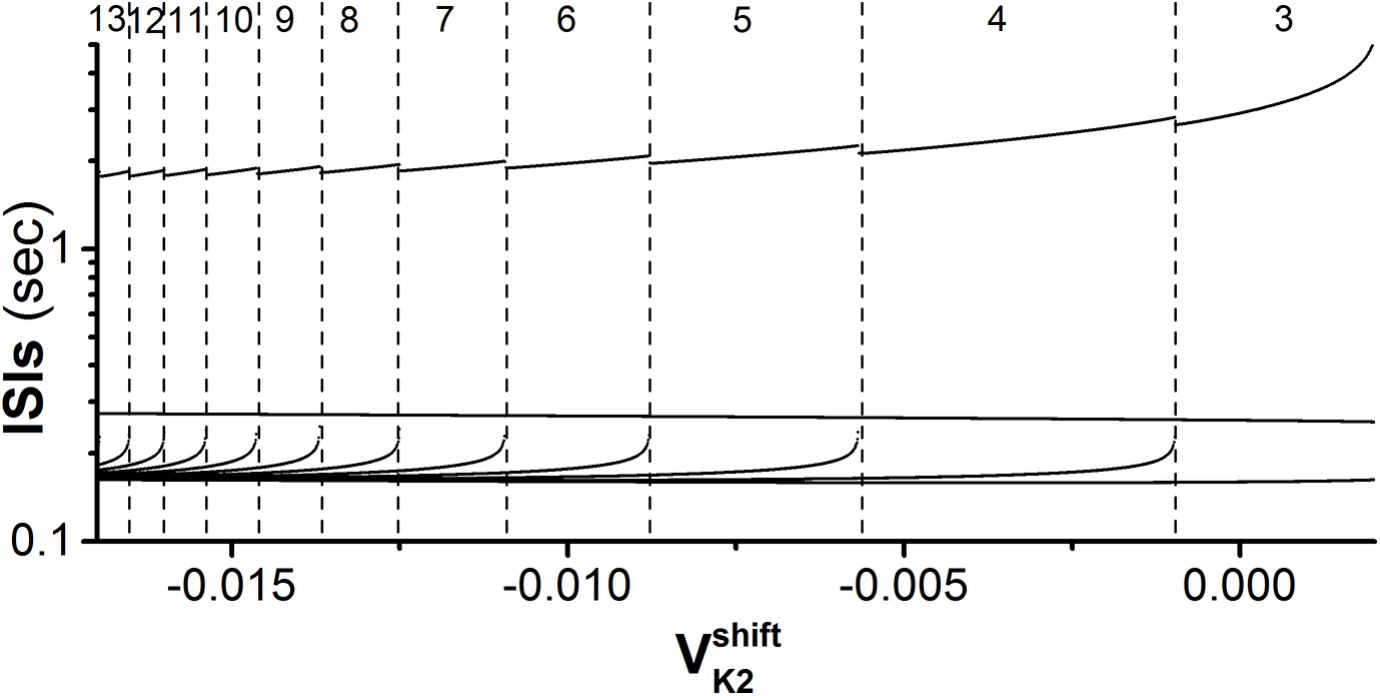
Period-adding bifurcation of interspike intervals (ISIs). The Leech heart interneuron model with −0.017 V < $V_{K2}^{shift}$ < 0.002 V. The numbers (from 3 to 13) lying between two dashed lines represent the number of spikes per burst of bursting pattern.

When the behavior of the neuron in the motif is chosen as period-5 bursting with $V_{K2}^{shift}$ = −0.008 V, the multiple synchronous behaviors appear within a period (2.778 s) of period-5 bursting of time delay at different coupling strength, as shown in Fig 11(A) (*g*_1_ = 0.2 nS and *g*_2_ = 0.16 nS), 11(B) (*g*_1_ = 0.3 nS and *g*_2_ = 0.18 nS), and 11(C) (*g*_1_ = 0.6 nS and *g*_2_ = 0.6 nS). If the bursting pattern of the motif is chosen as a bursting pattern within a bifurcation scenario with respect to $V_{K2}^{shift}$ (Fig 12, similar firing pattern has been investigated in Refs. [43, 44]), the motif can exhibit multiple synchronous behaviors similar to those of the present paper (not shown here).

## Discussion

Time delay induced multiple synchronous behaviors are simulated in inhibitory coupled neurons with endogenous bursting, which appear within a period of the endogenous bursting. This is different from the previous studies in which only one synchronous behavior appeared within a period of the endogenous firing, and the multiple synchronous behaviors appear when time delay is lengthened to a range of integer multiples of periods of the endogenous firing [27, 29]. In the present paper, when coupling strength is relatively small, only synchronous period-6 bursting that corresponds to the first of the multiple synchronous behaviors in the previous studies is induced at approximately half of the period of the endogenous bursting (Fig 8A and 8B). When coupling strength is relatively large, multiple synchronous behaviors are simulated when time delay is shorter than a period of endogenous bursting in inhibitory coupled neurons, which is a novel example of multiple synchronous behaviors.

We also examined other busting patterns with the same dynamics as the endogenous periodic bursting, such as period-5 bursting, and found that time-delay induced multiple synchronous behaviors can be found within a period of endogenous bursting. It shows that the multiple synchronous behaviors are independent to number of spikes per burst, but dependent to bursting. We also investigated multiple synchronous behaviors when time delay is lengthened to (*n*−1)*T* < *τ* < *nT* (*n* = 2, 3) (not shown here). Although not shown in the present paper, we examine the multiple synchronous behaviors simulated in network composed of Chay model with fold/homoclinic bursting or Rulkov map model with bursting. However, the multiple synchronous behaviors appear at time delay within a period of endogenous spiking has not been simulated in network composed of Hodgkin-Huxley model with spiking pattern. The results show that multiple synchronous behaviors are not dependent to the kind of neuronal model, but dependent to the kind of bursting. All these show that the multiple synchronous behaviors investigated in this paper are common in network composed of bursting neurons.

Furthermore, the bursting patterns of the synchronous behaviors, which are also dependent on time delay and coupling strength, show a close relationship with the endogenous period-6 bursting pattern. For example, the inhibitory coupled current with strong strength can terminate the behavior of period-6 bursting at *k* ^th^ spike to form the period-*k* (*k* = 4, 5), which can be well interpreted by the dynamics of endogenous period-6 bursting acquired with the fast-slow variable dissection method. With respect to the increase of time delay, the synchronous bursting patterns investigated in the present paper show a period-adding bifurcation from period-4 to period-7, which is similar to the previous studies wherein synchronous dynamics of the inhibitory coupled chaotic Hindmarsh-Rose neurons with distributed time delay are investigated [33]. However, the cause of the synchronous period-*k* (*k* = 1, 2, 3, 4, 5, 6) bursting patterns simulated at different time delays were not addressed in the investigation [33]. We suggest that the generation of these period-*k* (*k* = 1, 2, 3, 4, 5, 6) bursting patterns investigated in Ref [33] may obey the same rule as the synchronous behaviors of the present investigation. The present paper presents an example that different bursting patterns of the multiple synchronous behaviors of motif can be well explained by the dynamics of the endogenous bursting pattern of isolated neuron.

In the previous studies, synchronous behavior can be induced by inhibitory synapses with zero time delay in case of fast synapse at special conditions as investigated in Refs. [25, 26] and slow synapses [20–24] and inhibitory synapses with time delay [27–33]. The results of the present paper are different from these cases, presenting novel synchronous behaviors induced by inhibitory synapses with time delay. Our findings suggest that time delay and coupling strength are important for controlling or adjusting the novel synchronous bursting patterns. The results provide an inhibitory synaptic mechanism responsible for the generation of multiple synchronous behaviors in the nervous system. The period-6 bursting is identified as fold/homoclinic, which is a very common bursting pattern observed in different realistic nervous tissues or simulated neuronal models [39–41]. In addition, the motif composed of two coupled neurons with slow inhibitory synapses can achieve synchronous behavior, which has been demonstrated in the biological experiment [24]. The synchronous behavior is similar to the single synchronous behavior investigated in the present paper. If the coupling strength is increased, it is expected that the two coupled bursting neurons can achieve multiple synchronous behaviors, which should be demonstrated in biological experiment in future.
